# Advances in probiotics and gut microbiota-derived extracellular vesicles for the prevention and treatment of osteoporosis

**DOI:** 10.3389/fnut.2026.1884520

**Published:** 2026-10-19

**Authors:** Menghui Hu, Hui Li, Yaning Li, Hong Cao, Xiaoshuang Dai, Yan Tan, Qiuliyang Yu, Zhongxiang Wang, Xiaomin Wu, Jiake Xu, Xiaohua Pan

**Affiliations:** 1 Department of Biomedical Engineering, Southern University of Science and Technology, Shenzhen, Guangdong, China; 2 Faculty of Pharmaceutical Sciences, Shenzhen University of Advanced Technology, Shenzhen, Guangdong, China; 3 Institute of Biomedicine and Biotechnology, Shenzhen Institutes of Advanced Technology, Chinese Academy of Sciences, Shenzhen, Guangdong, China; 4 Shenzhen XBiome Biotech Co., Ltd., Shenzhen, Guangdong, China; 5 Department of Orthopaedics, The First Affiliated Hospital of Wenzhou Medical University, Wenzhou, China; 6 Institute of Clinical Translation and Regenerative Medicine, The Second Affiliated Hospital of Shenzhen University, Shenzhen, Guangdong, China; 7 School of Biomedical Sciences, University of Western Australia, Perth, WA, Australia

**Keywords:** EVs, gut microbiota, gut-bone axis, osteoporosis, probiotics

## Abstract

With the acceleration of population aging, the incidence of osteoporosis (OP) is increasing year by year. Although conventional pharmacological therapies remain the cornerstone of osteoporosis management, treatment selection and duration should be individualized according to fracture risk, treatment response, safety considerations, adherence, and the need for long-term monitoring. Emerging evidence underscores the gut microbiota’s role in bone homeostasis via the “gut-bone axis.” Probiotics, microbial metabolites (e.g., short-chain fatty acids), and their extracellular vesicles (EVs) exert dual effects: directly modulating osteoblast (OB) and osteoclast (OC) activity, and indirectly supporting bone health through gut-immune-endocrine multi-axis. Studies demonstrate that probiotics restore bone mass and microstructure in OP models. EVs derived from gut bacteria might act as mediators to regulate bone mass. Notably, synthetic biology-engineered EVs (e.g., BEVs-BMP-2-CXCR4) enhance bone-targeted therapeutic effects. However, the molecular mechanisms underlying EVs’ effects on OB and OC differentiation and activity remain unclear. This review focuses on the role of probiotics and probiotic extracellular vesicles in OP. Understanding the relationships between gut and bone in OP may provide an important framework for evaluating the therapeutic potential of probiotics and microbiota-derived EVs in OP.

## 1 Introduction

Osteoporosis (OP) is a major global public health problem and there is an urgent need to develop novel treatment strategies. OP is a systemic metabolic bone disease characterized by decreased bone mass and destruction of bone tissue microstructure. This leads to increased bone fragility and increased fracture risk. It is more common in postmenopausal women and the elderly (1). With the acceleration of the population’s aging process, the incidence of OP is increasing year by year. In 2016, the National Osteoporosis Foundation of the United States reported that as many as 1/2 of women and 1/4 of men aged 50 years or older would suffer from fractures due to OP, and studies have predicted that the number of OP patients in China will reach 212 million by 2050 (2, 3). According to the National Epidemiologic Survey of OP, the prevalence of OP in the population over 50 years old has reached 19.2%, and the prevalence of OP in the population over 65 years old has reached 32% (4). At the same time, OP and the follow-up medical treatment and care during the recovery period of bone fracture also impose a heavy economic burden on the family and society. Therefore, OP has become an urgent social and public health problem, and it is urgent to investigate its pathogenesis and develop novel treatment strategies.

With the development of molecular biology and microbiomics, recent studies have gradually revealed that gut microbiota is involved in the regulation of the “gut-bone axis,” which in turn affects bone health (5, 6). Gut microbiota influence bone homeostasis through multiple parallel pathways: directly via metabolites (e.g., short-chain fatty acids) and EVs, and indirectly through immune modulation and endocrine signaling. EVs of gut microbiota origin, in particular, have emerged as key mediators of inter-organ communication in this axis. Although several recent reviews have mapped the broader landscape of this field—such as Liu et al. (7) focusing on the biogenesis, isolation, and engineering of BEVs; Fang et al. (8) providing a panoramic framework of general EVs in osteoporosis; Liu et al. (9) proposing a three-tiered “gut-bone axis” including intestinal organoid-derived EVs (IOEVs); and Wei et al. (10) highlighting next-generation probiotics (NGPs) and engineered BEVs driven by machine learning. Instead, this is a narrative and critical review designed to provide four distinct incremental contributions: (1) a rigorous, critical appraisal of BEV safety—highlighting four frequently overlooked monitoring indicators (dynamic post-administration cytokine profiles, anti-EV neutralizing antibody responses, chronic RES accumulation toxicity, and microbiota perturbations) and emphasizing that engineered BEVs should not be assumed safe by default; (2) a granular cross-platform comparison of four engineering strategies (synthetic biology display, chemical conjugation, physical loading, and biomaterial-assisted delivery) detailing their respective trade-offs in Section 5.3.2 and Table 1; (3) an integration of the latest conflicting clinical trial evidence (11) showing that multi-strain Lactobacillus formulations failed to demonstrate protective effects in humans, thereby addressing the current translational discrepancies; and (4) a forward-looking, closed-loop roadmap (Figure 1) linking multi-omics, AI prediction, personalized intervention, bone-targeted delivery, and dynamic follow-up.

**Table 1:** Engineering strategies for EV-based osteoporosis therapy.

Engineering strategy	Representative modification	Therapeutic cargo/Target	Mechanism of action	Advantages	Limitations	Current status	References
Surface engineering + Synthetic biology	Recombinant *E. coli* Nissle 1917 expressing pET28a-ClyA-hCXCR4	CXCR4 (bone-targeting molecule)	CXCR4 displayed on EV surface enhances homing to SDF-1-rich bone tissue and improves tissue specificity	Enhanced bone-targeting ability; improved accumulation at osteoporotic lesions	No therapeutic cargo loaded; efficacy mainly depends on targeting enhancement; no clinical validation	Preclinical	(53)
Surface engineering + Cargo engineering + Synthetic biology	Recombinant *E. coli* Nissle 1917-pET28a-ClyA-BMP-2-CXCR4	BMP-2 + CXCR4	BMP-2 promotes osteogenic differentiation and bone formation; CXCR4 mediates bone targeting; activates BMP/SMAD signaling pathway	Simultaneous osteogenesis promotion and bone targeting; significantly ameliorates OVX-induced OP	Manufacturing complexity; safety, biodistribution and clinical translation remain unclear	Preclinical	(54)
Surface engineering + Cargo engineering	FRAT@BEV-DCS (FRAT-loaded BEV-DCS)	FRATtide (osteoclastogenesis-inhibitory peptide)	Targeted intracellular delivery of FRAT; regulates Akt/GSK-3β/NFATc1 signaling pathway; inhibits OC formation and bone resorption	Protects peptide from proteolytic degradation; excellent bone-targeting capacity; reduced toxicity and immunogenicity	No clinical studies; large-scale production and regulatory issues remain unresolved	Preclinical	(56)
Surface engineering + Peptide loading	BEV-DCS displaying DC-STAMP fusion protein on vesicle surface	Pre-OC-targeting DC-STAMP	DC-STAMP-mediated membrane fusion facilitates selective uptake by pre-OC	Specific targeting to OC precursors; enhanced intracellular delivery	Currently evaluated only in experimental models; long-term safety unknown	Preclinical	(56)
Chemical surface engineering	BT-LGG-EVs (bone-targeted LGG-EVs)	SDSSD bone-targeting peptide	Thiol-click conjugation of SDSSD peptide onto LGG-EVs enhances hydroxyapatite affinity and bone accumulation	Improved biodistribution and therapeutic efficacy	Peptide conjugation efficiency and long-term stability require optimization	Preclinical	(53)
Nucleic acid loading engineering	Bone-targeted BEVs delivering SOST-siRNA	SOST siRNA	Silencing SOST activates Wnt/β-catenin signaling and promotes bone formation	High gene-silencing efficiency and anabolic effect	siRNA stability and regulatory concerns	Preclinical	(55)
Physical engineering	Electroporation, sonication, extrusion, freeze–thaw cycles	miRNA, siRNA, proteins, small molecules	Physical disruption of EV membrane enables efficient cargo encapsulation	Simple, versatile, suitable for multiple cargo types	Possible EV membrane damage and cargo leakage	Preclinical	(59)
Hybrid engineering / Biomaterial-assisted engineering	EV-loaded hydrogels (GelMA, chitosan-collagen hydrogel), ECM-mimetic scaffolds	Pro-regenerative EVs	Sustained local release and enhanced retention of EVs at bone defects	Improved retention, controlled release, enhanced bone regeneration	Complex fabrication and scalability issues	Preclinical	(59)

*E. coli*, *Escherichia coli*; CXCR4, C-X-C Chemokine Receptor Type 4; SDF-1, Stromal Cell-Derived Factor 1; EV, Extracellular Vesicle; BMP-2, Bone Morphogenetic Protein 2; SMAD, Small Mothers Against Decapentaplegic; OVX, Ovariectomized; BEV, Bacterial Extracellular Vesicle; Akt, Protein Kinase B; GSK-3β, Glycogen Synthase Kinase 3 Beta; NFATc1, Nuclear Factor of Activated T-cells, Cytoplasmic 1; DC-STAMP, Dendritic Cell-Specific Transmembrane Protein; LGG, *Lactobacillus rhamnosus* GG; SDSSD, Ser-Asp-Ser-Ser-Asp (bone-targeting peptide sequence); SOST, Sclerostin; siRNA, Small Interfering RNA; miRNA, MicroRNA; GelMA, Gelatin Methacryloyl; ECM, Extracellular Matrix.

**Figure 1:**
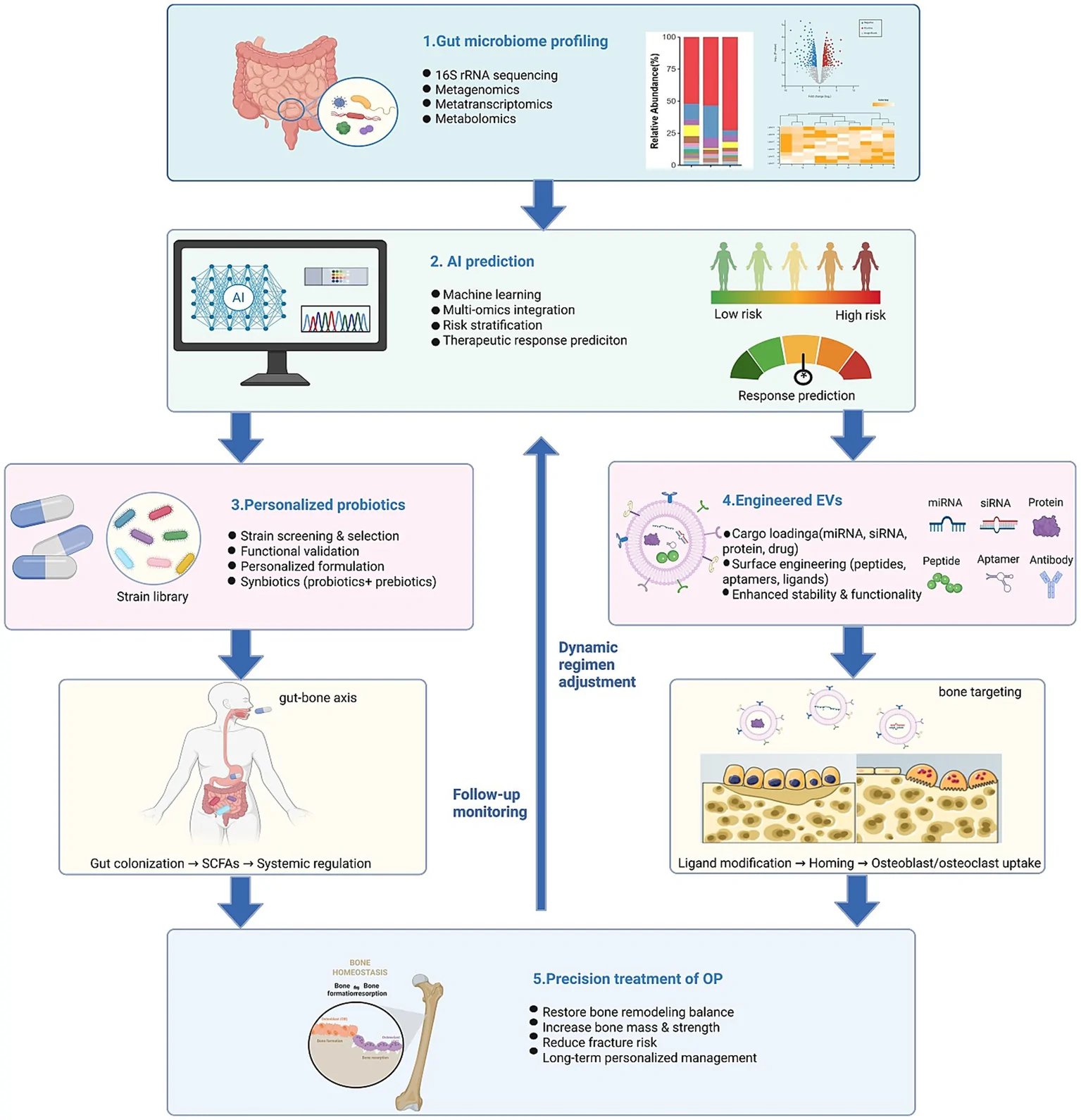
Schematic overview of the multi-step pipeline for precision treatment of OP via the gut-bone axis. This diagram illustrates the integrated workflow from multi-omics profiling and AI-based prediction to personalized intervention strategies (probiotics and engineered EVs), followed by bone-targeted delivery mechanisms and clinical endpoint management. The dashed feedback loop indicates that follow-up monitoring enables dynamic regimen adjustment through AI re-evaluation, forming a closed-loop precision medicine framework.

Literature searches for this narrative review were conducted in PubMed, Web of Science, and Scopus using keywords related to osteoporosis, bone remodeling, probiotics, gut microbiota, BEVs, microbial metabolites, bone-targeted therapy, bioengineering, and safety, supplemented by manual screening of reference lists. This review aims to synthesize representative mechanistic, preclinical, and clinical evidence while critically discussing the consistency, limitations, and translational implications of current findings. As this is not an exhaustive search, no PRISMA-compliant study screening or meta-analytic procedures were undertaken.

## 2 The limitations of medication for OP

Disruption of the dynamic balance (bone homeostasis) between OB-mediated bone formation and OC-mediated bone resorption is a key factor in the development of OP (12). Current therapeutic strategies are diversified, including basic therapy (calcium and vitamin D supplementation, exercise intervention), hormone therapy (estrogen supplementation, selective estrogen receptor modulators such as raloxifene, calcitonin, etc.), and pharmacological treatment. However, these therapies may carry side effects. The clinical efficacy, adverse effects, and long-term limitations of currently available anti-OP interventions are summarized in Table 2. Evidence for calcium and vitamin D supplementation in fracture prevention is limited and appears to vary by fracture site and population (13). Calcium supplementation-related risks include nephrolithiasis and, according to some meta-analyses, an increased incidence of cardiovascular events, although the studies included in these meta-analyses were not designed to assess cardiovascular outcomes (14). Estrogen use is restricted to a limited patient population and is associated with multiple potential adverse effects, including venous thromboembolism, stroke, myocardial infarction, cancers (breast, endometrial, ovarian), dementia, gallbladder disease, and urinary incontinence (15). Raloxifene does not significantly reduce the risk of hip or non-vertebral fractures and may cause venous thromboembolism, hot flashes, and leg cramps (16). Calcitonin similarly lacks a significant effect on reducing hip or non-vertebral fracture risk (13). For some therapies, particularly those whose skeletal benefits depend on continued treatment, discontinuation may be followed by substantial loss of BMD gains (17).

**Table 2:** Current pharmacological and non-pharmacological interventions for osteoporosis.

Therapeutic category	Representative drug/Intervention	Major clinical benefit	Main adverse effects/Limitations	Incidence or statistical data	Long-term use limitations	References
Calcium + Vit D	Calcium/Vit D	Maintains bone metabolism	Limited efficacy when used alone	BMD increase approximately 0.6–1.8%; fracture benefit remains limited/uncertain	Cannot reverse osteoporosis	(60)
Exercise	Resistance training	Improves balance and BMD	Efficacy depends on compliance	BMD increase of ~1–3%	Difficult to maintain long-term	(61)
HRT	Estrogen/HRT	↓Hip and vertebral fractures	Increased breast cancer risk	WHI study: breast cancer HR = 1.24	Only suitable for early postmenopausal women	(62)
SERM	Raloxifene	↓Vertebral fractures by 30–50%	Increased VTE risk	RR ≈ 1.4–3.0	Limited protection against hip fractures	(63)
Calcitonin	Calcitonin	Mildly increases BMD	Insufficient anti-fracture evidence	a large randomized trial found no significant reduction in vertebral, hip, or non-vertebral fractures	No longer a first-line therapy	(64)
Bisphosphonates	Alendronate, Risedronate, Zoledronic acid	Vertebral fracture reduction 40–70%; Hip fracture reduction 20–50%	Fever, headache, arthralgia; osteonecrosis of the jaw (ONJ); atypical femoral fractures (AFF)	ONJ: approximately 1–90 per 100,000 patient-years; AFF: approximately 3–50 per 100,000 patient-years	Long-term suppression of bone turnover; drug holiday required; cannot promote bone formation	(65, 66)
RANKL monoclonal antibody	Denosumab	FREEDOM trial: vertebral fracture reduction 68%; hip fracture reduction 40%	Hypocalcemia, infection risk, ONJ, AFF	Significant BMD decline within 12 months after discontinuation; 3- to 5-fold increased risk of multiple vertebral fractures	Requires long-term continuous administration; rebound bone loss after withdrawal	(67, 68)
Parathyroid hormone analog	Teriparatide	Vertebral fracture reduction 65%; non-vertebral fracture reduction 53%	Hypercalcemia, dizziness, nausea	FDA-approved maximum use: 24 months	Diminished efficacy after discontinuation; cannot fully repair bone microstructural damage	(69)
Anti-sclerostin monoclonal antibody	Romosozumab	FRAME trial: new vertebral fracture reduction 73%; ARCH trial: 48% reduction	Potential cardiovascular event risk	ARCH trial: cardiovascular events 2.5% vs. 1.9%	Insufficient long-term safety data; generally limited to 12 months of use	(70, 71)
Strontium salt	Strontium ranelate	↓ Vertebral fractures by 41%	Myocardial infarction (MI), VTE, DRESS syndrome	VTE: 0.9% vs. 0.6%; MI risk increased by ~60%	Restricted by EMA	(72)

Vit D, Vitamin D; BMD, Bone Mineral Density; WHI, Women‘s Health Initiative; HRT, Hormone Replacement Therapy; VTE, Venous Thromboembolism; RR, Relative Risk; RCT, Randomized Controlled Trial; ONJ, Osteonecrosis of the Jaw; AFF, Atypical Femoral Fractures; EMA, European Medicines Agency; DRESS, Drug Reaction with Eosinophilia and Systemic Symptoms; MI, Myocardial Infarction; FDA, U. S. Food and Drug Administration.

The development of drugs targeting the promotion of bone formation and inhibition of bone resorption has been a hot research topic in the field of OP prevention and treatment. Currently, anti-OP drugs are mainly categorized as inhibiting bone resorption and promoting bone formation. Avialalbe drugs that inhibit bone resorption (18) include: (1) bisphosphonates reduce bone resorption by inhibiting OC activity, but cause flu-like symptoms such as arthralgia, headache, and fever; long-term use may lead to osteonecrosis of the jaws and increase the risk of atypical fractures of the femur; and, their mechanism of action is only for the unidirectional inhibition of OC, and cannot promote bone formation. (2) RANKL monoclonal antibody (denozumab) blocks the RANKL activity to inhibit OC differentiation but may cause a rebound decrease in bone mineral density after stopping the drug. Bone formation-promoting drugs (18) include: (1) Parathyroid hormone analog (teriparatide): although it can directly stimulate OB proliferation, there is a therapeutic time window limitation (FDA-approved maximum use of 24 months). Moreover, it only temporarily increases bone mass without repairing bone microstructural damage. (2) Anti-sclerostin antibody (romosozumab): enhances OB activity by inhibiting Wnt pathway antagonists, but may cause cardiovascular events and has insufficient long-term safety data for postmenopausal OP. Despite its dual effects on both bone formation and resorption, the clinical use of strontium ranelate is restricted by cardiovascular events, venous thromboembolism, severe hypersensitivity reactions, and overestimation of bone mineral density (19).

The above data indicate that conventional pharmacotherapies remain the cornerstone of clinical management after OP diagnosis and have demonstrated robust efficacy in reducing fracture risk. However, their clinical use requires individualized consideration of treatment duration, adherence, contraindications, adverse-event profiles, and long-term monitoring. These considerations highlight the need for complementary strategies that may support bone health and address preventive needs, particularly among individuals at elevated risk of osteoporosis. In this context, the gut–bone axis has emerged as a promising complementary research avenue rather than a replacement for established pharmacotherapy. This bidirectional communication network between the gastrointestinal microbiome and the skeletal system integrates microbial, metabolic, immune, and endocrine signals to influence bone remodeling and systemic skeletal homeostasis.

## 3 The “gut-bone axis” plays an important role in the prevention and treatment of OP

Gut microbiota is a community of microorganisms—including bacteria, fungi, and viruses—that resides in the host gastrointestinal tract and regulates intestinal development, nutrient digestion, immune status, and, notably, bone homeostasis (20). Moreover, previous studies have provided evidence that the gut microbiome contributes to the regulation of the gut–bone axis. By characterizing gut microbiome alterations in postmenopausal women with OP, these studies identified taxa associated with OP and subsequently found that exogenous supplementation with the candidate strain *P. histicola* attenuated OVX-induced bone loss in mice (21). The main mechanism lies in the local microenvironmental regulation, *P. histicola* reduces intestinal permeability in estrogen-deficient mice by up-regulating the expression of tight junction proteins, including ZO-1 and Occludin, in the intestinal microenvironment; and the systemic regulation further prevents the secretion of pro-inflammatory factors by immune cells from being released into the circulatory system, thus indirectly inhibiting the differentiation and bone resorption function of OCs and preventing bone. This indirectly inhibits OC differentiation and bone resorption and prevents bone loss. This finding provides an experimental basis for the theory of “intestinal-bone axis,” reveals the potential value of the targeted intervention of gut microbiota in the prevention and treatment of OP, and opens up a new direction for the in-depth analysis of the molecular mechanism of OP and the development of new therapeutic strategies.

With the deepening of research and the development of science and technology, probiotics and their derivatives have been widely used in human health. Probiotics refer to a group of active microorganisms that, when ingested in sufficient quantities, can have a beneficial effect on the host’s health, including *bifidobacteria*, *lactobacilli*, *gram-positive cocci*, *yeasts*, and *bacilli,* etc., which can regulate the microecological balance of the intestinal tract, enhance immunity, and promote the absorption and utilization of nutrients (22). Studies have confirmed that specific probiotic strains can promote OB and inhibit OC differentiation and function, which can synchronously improve the bone microstructure and calcium and phosphorus metabolism balance, thus maintaining bone homeostasis, and providing a new microbial-targeted intervention strategy for the prevention and treatment of OP (23, 24). Therefore, it is important to explore probiotic strains with anti-OP properties and elucidate their molecular mechanisms to regulate the “gut-bone axis,” so as to lay a translational foundation for the construction of microbial-host co-metabolism intervention strategies, which will become an important breakthrough direction in the field of OP prevention and treatment.

## 4 Current research on probiotics and their derivatives in the prevention and treatment of OP

The gut microbiota participates in the regulation of bone homeostasis through the “gut-bone axis,” positioning probiotics and their bioactive derivatives as an emerging safe intervention direction for OP. Accumulating evidence indicates that probiotics exert bone-protective effects through two synergistic pathways: (1) the direct pathway—viable probiotics, soluble metabolites, and bacterial EVs directly act on OBs and OCs to bidirectionally modulate bone remodeling; (2) the indirect pathway—by repairing intestinal barrier integrity, reshaping immune homeostasis, and modulating endocrine signaling, probiotics systemically reduce bone resorption. In addition, prebiotics—dietary substrates that selectively promote probiotic proliferation—can also improve calcium absorption and support bone health.

This nutraceutical paradigm occupies a distinctive position within the OP management spectrum. Unlike conventional pharmacotherapies—which are typically initiated after diagnosis, limited by safety windows and side-effect profiles, and often requiring discontinuation after a defined period—nutraceutical interventions, including probiotics, prebiotics, postbiotics, and fermented foods, can be integrated into daily dietary patterns for primary prevention in at-risk populations. Their generally favorable tolerability profiles, accessibility, and potential compatibility with dietary patterns make them attractive candidates for long-term, population-level bone health maintenance, although their relatively modest and variable efficacy requires realistic expectation management. Importantly, nutraceuticals should not be viewed as replacements for pharmacotherapy in established OP, but rather as complementary strategies that may extend the preventive window and reduce the overall treatment burden.

Before detailing the regulatory mechanisms, it is useful to outline the organizational logic of this section. We first summarize the direct and indirect pathways of probiotic strains and their derivatives at the cellular and animal levels (Sections 4.1 and 4.2), then compile human clinical evidence (Section 4.3), and finally discuss the inherent limitations of natural probiotic systems, which sets the stage for the engineered EV strategies covered in Section 5 (Section 4.4).

### 4.1 Direct regulatory effects of probiotics and their derivatives on bone metabolism

Probiotics and their secreted metabolites and EVs can directly target bone-lineage cells to promote osteogenesis and inhibit OC differentiation, respectively. The relevant *in vitro* cellular and *in vivo* animal model evidence is summarized in Tables 3, 4.

**Table 3:** Research on the improvement of OP by probiotics.

Probiotics	Sources	*In vitro*	*In vivo*	References
*Prevotella histicola*	BeNa Biological Company	——	Alleviates OP symptoms, reduces intestinal inflammation, and repairs intestinal mucosal barrier damage	(73)
*Bifidobacterium longum*	human	——	Improves bone damage; regulates serum bone metabolism marker levels, promotes bone formation, inhibits bone resorption, and regulates the gut microbiota	(74)
*Bifidobacterium longum* 35624®	Precision Biotics Ltd	*Bifidobacterium longum* 35624® extra-surface polysaccharide-mediated inhibition ofOCt formation is TLR2-dependent	Oral gavage partially prevents bone loss in OVX mice	(28)
*Bifidobacterium pseudocatenulatum* CECT 7765	Human	——	Increased serum OCN, increased Wnt/β-catenin pathway gene expression, decreased serum C-terminal peptide (CTX) and parathyroid hormone (PTH)	(75)
*Akkermansia muciniphila*	Human	EVs are major effectors of CGM and Akk in the regulation of bone metabolism	Direct supplementation with Akk significantly improved the OP phenotype in OVX mice, and direct oral administration of CGM-EVs and Akk-EVs significantly increased bone mass, bone length, and bone strength.	(20)
*Lactobacillus helveticus* (ATCC 4356)	ATCC	——	Significantly increased serum levels of bone formation markers (e.g., OC), decreased serum levels of bone resorption markers (e.g., CTX), improved bone microarchitecture, increased bone mineral density and femur strength, and up-regulated the expression of osteogenesis-related genes (e.g., Runx2 and Bmp2)	(76)
*Bifidobacterium longum*	ATCC	——	*B. longum* increases BMD by up-regulating the expression of Sparc and Bmp-2 genes, altering the microstructure of the femur	(77)
*Lactobacillus rhamnosus* GG	Human	——	Improves bone microstructure and biomechanical properties, regulates the Th17/Treg balance, improves intestinal barrier function, and regulates bone metabolism - related indexes	(30)
*Lactobacillus rhamnosus* GG	Human	——	Enhancement of intestinal barrier integrity and prevention of bone loss due to sex hormone deficiency	(34)
*Lactobacillus reuteri* ATCC PTA 6475	ATCC	Inhibits osteoclastogenesis induced by RANKL and M - CSF by 70%	Inhibits bone loss, alters the gut microbial community structure, and modulates the expression of bone metabolism - related factors	(26)
*Lactobacillus reuteri* ATCC PTA 6475	ATCC	——	Reduction of bone loss in antibiotic-treated mice, up-regulation of femur and vertebrae BV/TV, BMD, BMC, Tb. N., MAR, serum OCN	(78)
*Lactobacillus fermentum* ZS40	Yak yogurt	——	Improve serum biochemical indicators related to bone metabolism, up-regulate the expression of genes related to bone formation, inhibit the formation of OC, and significantly increase bone density.	(79)
*Lactobacillus acidophilus* ATCC 4,356	ATCC	LA inhibits bone loss by modulating Treg-Th17 cell homeostasis in mice with significantly reduced levels of IL-6, IL-17A, and TNF-α and significantly increased levels of IFN-γ and IL-10	Inhibit bone loss, improve bone microstructure and increase bone density.	(80)
*Propionibacterium freudenreichii* MJ2	raw milk	Promotes OB differentiation and mineralization, activates the BMP2/RUNX2 signaling pathway	No hepatotoxicity, increases bone mineral density (BMD), and inhibits bone loss	(81)
*Lactobacillus reuteri* ATCC PTA 6475	BioGaia AB (human)	——	Significant reduction in total volumetric tibial bone mineral density loss in older women with low bone mineral density	(40)
*Lactobacillus paracasei* DSM 13434, *Lactobacillus plantarum* DSM 15312 和 *Lactobacillus plantarum* DSM 15313	Probi (human)	——	Protects against bone loss in mice, probiotic treatment significantly reduces lumbar bone density loss in early postmenopausal women in human clinical trials	(38)

ATCC, American Type Culture Collection; TLR2, Toll - like receptor 2; CGM, Conjugated Linoleic Acid Glycerides; RANKL, Receptor Activator of Nuclear Factor - κB Ligand; M - CSF, Macrophage Colony - Stimulating Factor; Th17, T helper 17 cell; Treg, Regulatory T cell; IL - 6, Interleukin - 6; IL - 17A, Interleukin - 17A; TNF - α, Tumor Necrosis Factor - alpha; IFN-γ, Interferon - γ; IL - 10, Interleukin - 10; OB, Osteoblast; BMP2, Bone Morphogenetic Protein 2; RUNX2, Runt - related transcription factor 2; OVX, Ovariectomized; OCN, Osteocalcin; BMD, Bone Mineral Density; BV/TV, Bone Volume/Tissue Volume; BMC, Bone Mineral Content; Tb. N, Trabecular Number; MAR, Mineral Apposition Rate.

**Table 4:** Efficacy of probiotic metabolites in the prevention and treatment of OP.

Probiotic metabolites	Sources	*In vitro*	*In vivo*	References
Extracellular Vesicles (EVs)	*Akkermansia muciniphila*	Promotes the expression of Bglap, Alpl, and Runx2 and inhibits the expression of Trap, Ctsk, Mmp9, Oc - stamp, and Oscar	Promotes osteoblastogenesis and inhibits osteoclastogenesis; increases new bone formation and mineralization	(20)
Serum Valproic Acid	*Oscillibacter valericigenes* and *Megasphaera elsdenii*	Inhibits the inflammatory response and increases ALP activity and extracellular matrix mineralization	Inhibits bone resorption and prevents the deterioration of bone structure in OVX mice	(82)
Short - chain Fatty Acids (SCFA)	*Prevotella*	Promotes the shift of OC precursor metabolism to glycolysis, triggers cellular stress, and inhibits OC differentiation	Significantly lower serum levels of CTX - I, reduced OC counts, prevents bone loss, and reduces the inflammatory response	(32)
Melatonin (MLT)	*Roseburia hominis*	Shifts macrophages to the M2 phenotype	MLT is significantly correlated with the BMD value and can effectively improve OVX - induced bone loss	(33)
Butyric Acid (Butyrate, C4)	*Lactobacillus acidophilus*	Inhibits the expression of CTSK, Acp5, and c - Fos and inhibits OC formation	Improves bone structure, improves intestinal permeability, inhibits bone resorption, and inhibits RANKL expression	(31)

Bglap, Bone Gla - protein; Alpl, Alkaline Phosphatase; Runx2, Runt - related transcription factor 2; Trap, Tartrate - resistant acid phosphatase; Ctsk, Cathepsin K; Mmp9, Matrix metalloproteinase 9; Oc - stamp, Osteoclast - associated immunoglobulin - like receptor; Oscar, Osteoclast - associated receptor; ALP, Alkaline Phosphatase; Acp5, Acid phosphatase 5; c - Fos, cellular homolog of FBJ murine osteosarcoma viral oncogene; OVX, Ovariectomized; CTX - I, C - terminal Telopeptide of Type I Collagen.

#### 4.1.1 Direct bone-protective effects of viable probiotic strains

Multiple *Lactobacillus*, *Bifidobacterium*, and *Akkermansia* strains have been demonstrated to directly modulate osteoblastic and osteoclastic activity. Based on their functional characteristics, these strains can be broadly classified into three categories: (1) osteogenesis-promoting types—for example, *Lactobacillus rhamnosus GG* promotes bone formation through a butyrate-dependent, Treg-mediated mechanism involving WNT10B signaling (25); (2) bone-resorption-inhibiting types—for instance, *Lactobacillus reuteri* ATCC PTA 6475 inhibits approximately 70% of RANKL-induced OC precursor differentiation *in vitro* and significantly ameliorates OVX-induced bone loss in mice (26); (3) mixed-function types—such as *Bifidobacterium longum*, which both inhibits RANKL-induced osteoclastogenesis and increases bone volume and strength in mice (27). Furthermore, *Bifidobacterium longum* subsp. 35624® inhibits OC formation via TLR2-dependent signaling mediated by its surface exopolysaccharide EPS624 (28), while *Akkermansia muciniphila* derived from the pediatric gut microbiota exerts bone-protective effects through the release of EVs that promote bone formation and inhibit bone resorption (20). Notably, the efficacy of different strains exhibits marked species-specificity: for example, a mixed formulation of *Lactobacillus paracasei* and *Lactobacillus plantarum* significantly delays lumbar spine trabecular bone loss in early postmenopausal women but shows no protective effect on hip cortical bone (29), suggesting that site-specific effects require further mechanistic investigation (Table 3).

It is noteworthy, however, that contradictory findings exist even for the same probiotic strain. For example, *Lactobacillus rhamnosus* GG has been reported to promote bone formation via butyrate-driven Treg-mediated WNT10B expression in one study (25), while another study emphasized its role in restoring Th17/Treg balance without detecting significant changes in butyrate levels (30). These discrepancies may arise from differences in mouse strains, dosing regimens, baseline microbiota composition, or the timing of intervention relative to OVX. Such inconsistencies underscore the critical need for standardized experimental protocols and head-to-head comparisons across independent laboratories.

#### 4.1.2 Direct regulation of bone remodeling by soluble probiotic metabolites

Short-chain fatty acids (SCFAs), valeric acid, melatonin, and other bioactive small molecules produced by probiotic fermentation are key soluble mediators of the gut-bone axis (Table 4). Butyrate directly suppresses the expression of OC-related genes such as CTSK and c-Fos, thereby inhibiting OC formation (31). SCFAs reduce osteoclastogenesis by shifting the glucose metabolism of OC precursors (32). Microbiota-derived melatonin improves bone microarchitecture in OVX mice (33). Notably, these metabolites often simultaneously exert intestinal barrier-repairing functions, creating a linked “gut repair–bone protection” effect. For example, butyrate not only suppresses osteoclastic activity but also reduces intestinal permeability by enhancing tight junction protein expression, thereby indirectly attenuating systemic inflammation.

#### 4.1.3 Direct regulatory functions of gut microbiota-derived EVs

In addition to soluble metabolites, gut microbiota-derived extracellular vesicles (BEVs) can serve as signaling mediators that reach bone tissue. Natural BEVs carry proteins, nucleic acids, and other bioactive molecules and have been shown to regulate osteogenic and osteoclastogenic gene expression *in vitro*, with bone-mass improvement also observed in animal studies. For example, *Akkermansia muciniphila*-derived EVs upregulate osteogenic genes (Runx2, Alpl) and suppress osteoclastogenic genes (Trap, Ctsk), and oral administration of these vesicles significantly increases bone mass in OVX mice (20). However, natural BEVs lack bone-targeting capacity, and their bone-tissue enrichment following systemic administration remains inefficient, severely limiting their therapeutic efficacy *in vivo*—a limitation that will be critically addressed through engineering strategies in Section 5.

### 4.2 Indirect systemic pathways through which probiotics regulate bone metabolism

Beyond their direct effects on bone cells, probiotics also maintain bone homeostasis indirectly through the gut, immune, and endocrine axes (Figure 2). These pathways should not be interpreted as independent or strictly linear routes. Intestinal barrier integrity, immune signaling, microbial metabolites, and endocrine regulation are dynamically interconnected, and the same probiotic intervention may influence several nodes simultaneously. Accordingly, associations between a single metabolite or signaling pathway and a bone phenotype should be distinguished from causal evidence, which requires targeted perturbation and mechanistic validation. Current evidence indicates that indirect regulation operates primarily through the following three pathways:

(1) Repair of the intestinal mucosal barrier: Probiotics such as LGG and *Lactobacillus plantarum* upregulate the expression of tight junction proteins ZO-1 and Occludin in intestinal epithelial cells, thereby reducing intestinal permeability and limiting the translocation of pro-inflammatory substances such as lipopolysaccharide (LPS) into the circulation, consequently blocking the transmission of inflammatory signals to the skeletal system. For example, LGG supplementation in estrogen-deficient mice significantly improves intestinal barrier function and prevents bone loss (34).
(2) Reshaping of immune homeostasis: Probiotics modulate the Th17/Treg cell ratio, reducing the secretion of pro-inflammatory cytokines such as IL-6 and TNF-α and thereby attenuating inflammation-mediated OC overactivation. *Lactobacillus* intervention significantly reduces serum TNF-α and IL-17A levels while increasing the anti-inflammatory cytokine IL-10 in OVX mice (35).
(3) Endocrine and metabolic regulation: Probiotics enhance intestinal SCFA levels, modulating Wnt/β-catenin signaling and parathyroid hormone-related pathways to improve calcium and phosphorus absorption and utilization. For instance, the chicken-derived probiotic *Anaerostipes caccae* CML199 promotes osteogenic differentiation and delays aging-related bone loss through butyrate-mediated improvement of the bone marrow immune microenvironment (36).

**Figure 2:**
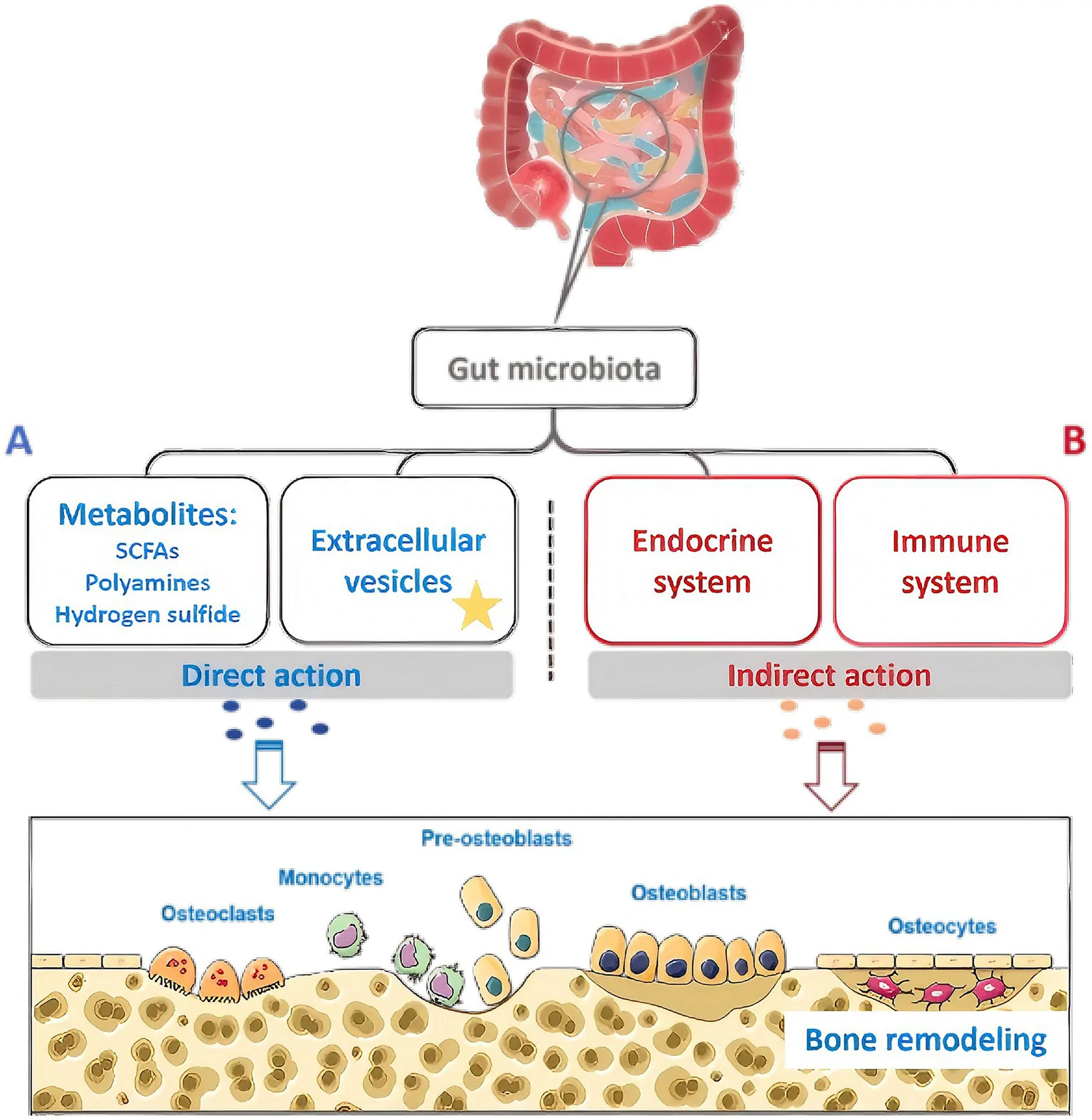
Gut microbiota regulate bone remodeling through direct and indirect pathways. This is mainly reflected in: **(A)** Gut microbiota directly affect the differentiation and function of OB and OC through their metabolites (e.g., short-chain fatty acids) and secretion of EVs; **(B)** at the same time, Gut microbiota also indirectly affect the differentiation and function of OB and OC by regulating the secretion of hormones (e.g., estrogens) and the secretion of proinflammatory/inflammation inhibitory factors by immune cells (e.g., Th17/Treg).

Multiple animal studies have shown that butyrate-related metabolic pathways can contribute to the bone-protective effects of probiotics (25, 36, 37), indicating that the gut immune-endocrine axis constitutes a critical intermediary pathway in probiotic-mediated bone protection.

### 4.3 Human clinical evidence for probiotics and prebiotics

The mechanistic studies at the cellular and animal levels provide biological plausibility for the bone-protective effects of probiotics; however, the translational value of these interventions ultimately requires validation through human clinical trials. To date, several randomized controlled trials (RCTs) have evaluated the effects of probiotics and prebiotics on bone metabolism markers and bone mineral density, as summarized in Table 5.

**Table 5:** Clinical studies of probiotics, prebiotics, and related interventions in osteoporosis and bone metabolism.

Intervention	Population	Design	Main Findings	Evidence Level / Clinical Relevance	Limitations	References
*Bifidobacterium lactis* Probio-M8	Postmenopausal women aged 50–80 years; DXA T-score ≤ − 1.0 with FRAX fracture risk ≥7%, or T-score ≤ − 2.5; diagnosed with postmenopausal osteoporosis (PMO)	Single-center, randomized, double-blind, placebo-controlled pilot trial	Probio-M8 group showed tsignificantly increased serum vitamin D₃, and decreased PTH, serum calcium, procalcitonin, and osteocalcin; enriched SCFA-producing bacteria, strengthened beneficial microbial symbiotic network; significantly upregulated gut carbohydrate and phospholipid metabolism-related functional genes	Level 1b; direct osteoporosis-related clinical evidence, but limited by small sample size and short intervention duration	Small sample size; only 3-month intervention with no observed BMD improvement; even smaller sample in fecal sequencing subgroup; no metabolomics data, limiting causal inference between microbiota and bone metabolism	(83)
Multi-strain probiotic capsules (*Lactobacillus casei*, *Bifidobacterium longum*, *Lactobacillus acidophilus*, *Lactobacillus rhamnosus*, *Lactobacillus bulgaricus*, *Bifidobacterium breve*, and *Streptococcus thermophilus*)	Iranian women aged 50–72 years with postmenopausal osteopenia (T-score −1.0 to −2.5)	Single-center, randomized, double-blind, placebo-controlled clinical trial	No significant improvement in lumbar spine or total hip BMD; probiotic group showed significant reductions in bone turnover markers BALP and CTX, as well as decreased PTH and pro-inflammatory cytokine TNF-α, indicating suppressed bone resorption	Level 1b; direct osteoporosis-related clinical evidence, with effects mainly observed in bone turnover markers rather than BMD	Limited total sample size; only baseline and 6-month time points with no intermediate dynamic observations; only serum biomarkers measured; intervention duration only 6 months	(39)
Composite probiotic (*Lactobacillus reuteri* GL-104, *Lactobacillus paracasei* MP-137, *Lactobacillus rhamnosus* MP108, *Lactobacillus rhamnosus* F-1, *Lactobacillus rhamnosus* BV77, *Bifidobacterium animalis* ssp. lactis CP-9, *Bifidobacterium longum ssp. longum* OLP-01, *Bacillus coagulans*)	Postmenopausal osteopenic women aged 45–70 years (lumbar spine/hip T-score −2.5 to −1.0)	Single-center, randomized, double-blind, placebo-controlled clinical trial	Baseline CTX levels were significantly higher in the probiotic group than in the placebo group; after 12 weeks of intervention, CTX decreased significantly from baseline in the probiotic group (*p* = 0.026) but not in the placebo group; the change in CTX between groups was significantly different (*p* < 0.001)	Level 1b; direct bone-metabolism clinical evidence, with evidence limited to short-term changes in CTX	Small sample size; baseline CTX imbalance between groups may confound result interpretation; only 12-week intervention with short follow-up; no BMD measurement, relying solely on biochemical markers	(84)
*Lactobacillus reuteri* ATCC PTA 6475	90 elderly women with low BMD	Randomized double-blind placebo-controlled trial	Reduced tibial bone loss by ~50% compared with placebo	Level 1b; direct bone-loss-related clinical evidence	Small sample size; only tibial BMD benefit observed, with no statistically significant differences in spine or hip parameters; gut microbiota not analyzed, limiting mechanistic insights; only 12-month follow-up, lacking long-term BMD and fracture endpoint data	(40)
*Limosilactobacillus fermentum* CCFM1126	Patients aged ≥45 years with osteoporosis or osteopenia (both sexes); 17 in probiotic group, 14 in placebo group (final)	Randomized, double-blind, placebo-controlled parallel trial (Chinese Clinical Trial Registry: ChiCTR2300076115)	CCFM1126 significantly increased BMD (T-score change: +0.39 vs. − 0.39 in placebo, *p* = 0.0047; Z-score change: +0.41 vs. − 0.36, *p* = 0.0059); serum calcium (*p* = 0.0226) and vitamin D (*p* = 0.0040) significantly elevated; OPG increased (*p* = 0.0206) and TRACP decreased (*p* = 0.0079); gut microbiota remodeled (Lactobacillus and Weissella increased; Paraprevotella, Marvinbryantia, Ruminococcus 1 decreased)	Level 1b; direct osteoporosis/osteopenia-related clinical evidence, but preliminary due to small sample size and short duration	Small sample size (only 31 completers); short intervention duration (3 months); no multi-dose comparison; findings require validation in larger cohorts	(41)
Composite *Lactobacillus* formulation (*Lactobacillus paracasei* DSM 13434, *Lactobacillus plantarum* DSM 15312, *Lactobacillus plantarum* DSM 15313)	249 healthy early postmenopausal women	Multicenter, randomized, double-blind, placebo-controlled clinical trial	Composite Lactobacillus significantly prevented lumbar spine trabecular bone loss in early postmenopausal women, with greater benefit in those within 6 years of menopause; no protective effect on hip cortical bone; no intergroup differences in bone-related or inflammatory markers; formulation was safe and well-tolerated	Level 1b; direct bone-loss-related clinical evidence relevant to osteoporosis prevention	Single dose regimen; only 12-month short-term intervention; homogeneous study population (healthy early postmenopausal women, not osteoporotic patients); no key gut metabolites measured, limiting mechanistic interpretation; inability to predict efficacy through blood biomarkers; site-specific (vertebral vs. hip) effects remain unexplained	(38)
*Lactiplantibacillus plantarum* DSM15312 + *L. plantarum* DSM15313 + *Lactisaceibacillus paracasei* DSM13434	114 early postmenopausal females (1–8 years since menopause), aged 45–65 years, with lumbar spine or total hip T-score > − 2.5; 58 in probiotic group, 56 in placebo group	Multicenter, randomized, double-blind, placebo-controlled trial (ACTRN12621000810819)	The primary outcome showed a greater decline in distal tibia total vBMD in the probiotic group than in the placebo group (between-group difference: −3.7 mgHA/cm^3^, 95% CI: −6.9 to −0.4, *p* = 0.027); radial total vBMD also showed a greater decline in the probiotic group (*p* = 0.044, not significant after multiplicity adjustment); lumbar spine and forearm aBMD declined more in the probiotic group (not significant after adjustment); no significant between-group differences were observed in bone turnover markers, calciotropic hormones, or inflammatory markers; the probiotic did not show bone-protective effects and was associated with greater bone loss	Level 1b; high-quality direct bone-loss/BMD-related clinical evidence, with findings inconsistent with the earlier positive RCT	Primary outcome not adjusted for multiple comparisons; baseline imbalances in dietary calcium intake and walking time between groups; magnitude of changes small (~1–3%) and within measurement COV; self-reported dietary and physical activity data subject to bias; generalizability limited to early postmenopausal females; 12-month duration may be insufficient for cortical changes	(11)
Galacto-oligosaccharides (GOS)	Adolescent girls	Randomized double-blind crossover trial	5 g/d GOS significantly increased calcium absorption and Bifidobacterium abundance; 10 g/d showed diminished effect with no dose–response relationship; peak calcium absorption occurred at 24–36 h (characteristic of colonic absorption); mild gastrointestinal side effects; greater benefit observed in individuals with low baseline calcium absorption	Level 1b; clinical evidence for calcium absorption and gut microbiota modulation; indirect evidence for osteoporosis	Small sample size; only white adolescent girls, limiting generalizability; only 3-week intervention with no long-term BMD follow-up; no SCFA measurement in the gut, precluding complete mechanistic elucidation of GOS-enhanced calcium absorption	(42)
Inulin-type fructans	Adolescents	Randomized double-blind parallel controlled trial	8-week and 1-year fructan supplementation both significantly increased calcium absorption; after 1 year, greater gains in whole-body bone mineral content and BMD; gene-interaction effect observed, with FF/Ff genotype individuals showing more pronounced benefit; only 1 participant experienced mild gastrointestinal intolerance	Level 1b; clinical evidence for calcium metabolism and general skeletal health; indirect evidence for osteoporosis	No dose gradient of fructans tested, preventing identification of optimal intake; no SCFA measurement, limiting mechanistic understanding of calcium absorption enhancement; no evaluation under very low calcium diet conditions; study population limited to adolescents	(43)

DXA, Dual-energy X-ray Absorptiometry; FRAX, Fracture Risk Assessment Tool; PMO, Postmenopausal Osteoporosis; PTH, Parathyroid Hormone; SCFA, Short-Chain Fatty Acid; BMD, Bone Mineral Density; RCT, Randomized Controlled Trial; BALP, Bone-specific Alkaline Phosphatase; CTX, C-terminal Telopeptide of Type I Collagen; TNF-α, Tumor Necrosis Factor-alpha; ATCC, American Type Culture Collection; GOS, Galacto-oligosaccharides.

#### 4.3.1 Composite probiotic formulations

A multicenter RCT of a triple-Lactobacillus formulation (*Lactobacillus paracasei* DSM 13434, *Lactobacillus plantarum* DSM 15312, and *Lactobacillus plantarum* DSM 15313) in 249 healthy early postmenopausal women demonstrated that this formulation significantly delayed lumbar spine trabecular bone loss (38), with greater benefit observed in women within 6 years of menopause; however, no protective effect on hip cortical bone was observed, and no intergroup differences in bone-related or inflammatory markers were detected. Importantly, a recently published RCT evaluating the identical three-strain Lactobacillus formulation failed to replicate the bone-protective effects (11) reported by Jansson et al. In this 12-month trial involving 114 early postmenopausal women, the probiotic group exhibited a greater decline in distal tibia volumetric BMD compared with placebo (between-group difference: −3.7 mgHA/cm^3^, 95% CI: −6.9 to −0.4, *p* = 0.027), with no significant improvements in bone turnover markers or inflammatory cytokines. These discordant findings underscore the notion that the osteoprotective efficacy of probiotics is likely context-dependent, influenced by baseline nutritional status, gut microbiota composition, and host genetic factors. Accordingly, the clinical evidence for this specific three-strain formulation should be considered mixed rather than uniformly positive, and further adequately powered trials are warranted before probiotics can be recommended as a standalone intervention for skeletal preservation in postmenopausal women.

Another 7-strain probiotic capsule trial in Iranian postmenopausal women with osteopenia failed to show significant improvement in lumbar spine or total hip BMD, although it did reduce bone resorption markers (BALP, CTX) and the pro-inflammatory cytokine TNF-α (39).

#### 4.3.2 Single strains

An RCT of *Lactobacillus reuteri* ATCC PTA 6475 in 90 elderly women with low BMD demonstrated that this strain reduced tibial bone loss by approximately 50% compared with placebo, but no statistically significant differences were observed in spinal or hip parameters (40). A recent randomized, double-blind, placebo-controlled trial found that 3-month supplementation with Limosilactobacillus fermentum CCFM1126 improved T-score and Z-score, accompanied by favorable changes in serum calcium, vitamin D, OPG, TRACP and gut microbiome composition (41).

#### 4.3.3 Prebiotics

Clinical studies of prebiotics have primarily evaluated calcium absorption and general skeletal health rather than osteoporosis-specific treatment outcomes. A crossover trial of galacto-oligosaccharides (GOS) in adolescent girls showed that 5 g/d GOS significantly increased calcium absorption and *Bifidobacterium* abundance, whereas 10 g/d showed diminished effects without dose-responsiveness (42); peak calcium absorption occurred at 24–36 h post-administration, consistent with colonic absorption characteristics. A parallel controlled trial of inulin-type fructans in adolescents demonstrated that both 8-week and 1-year fructan supplementation significantly increased calcium absorption, with greater gains in whole-body bone mineral content and BMD after 1 year (43). These findings provide clinical evidence that prebiotics can modulate calcium absorption and parameters related to skeletal health; however, because these studies were conducted primarily in adolescent populations and did not specifically evaluate osteoporosis prevention or treatment, they should not be interpreted as direct evidence of therapeutic efficacy against osteoporosis. Direct clinical evidence in populations at risk of osteoporosis or with established osteoporosis remains limited.

#### 4.3.4 General limitations of clinical studies

Most trials suffer from small sample sizes and short intervention durations (typically 3–12 months), relying on blood biochemical markers rather than long-term BMD follow-up or fracture endpoint data. The study populations are often homogeneous (predominantly white adolescent girls or healthy early postmenopausal women), limiting generalizability to osteoporotic patients and diverse populations. Moreover, most studies have not measured key gut metabolites, precluding complete elucidation of the causal relationship between microbiota modulation and bone metabolism.

### 4.4 Summary and perspectives

Collectively, cellular, animal, and human studies demonstrate that probiotics can ameliorate OP through two complementary pathways: direct regulation of bone cell differentiation and function, and indirect modulation of the gut-immune-endocrine network. Viable probiotics, soluble metabolites, and natural EVs all exhibit bone-protective activity.

However, natural probiotic systems face three inherent limitations: (1) high strain-specific heterogeneity—the bone-protective efficacy varies markedly across different species and even among strains of the same species, with the underlying mechanisms poorly understood; (2) lack of bone-targeting capacity in natural EVs—poor bone-tissue distribution following systemic administration results in a narrow therapeutic window; and (3) insufficient clinical evidence—most RCTs have small sample sizes and short durations, with a lack of long-term efficacy and safety data, in addition to a significant risk of publication bias (underreporting of negative results).

To overcome these bottlenecks, researchers are developing bone-targeting engineered BEVs using synthetic biology, chemical conjugation, physical loading, and biomaterial-assisted delivery platforms to achieve precise delivery to bone tissue. The next section will critically discuss the core value of microbiota-derived EVs in the gut-bone axis and review various engineering strategies and their translational prospects.

## 5 Critical role of gut microbiota-derived EVs in the “gut-bone axis”

EVs derived from the gut microbiota are increasingly recognized as mediators of intercellular and interkingdom communication along the gut–bone axis. EVs are cell-released extracellular particles delimited by a lipid bilayer and incapable of autonomous replication. They are produced by diverse organisms, including mammals, plants and bacteria, and can transport proteins, nucleic acids, lipids, metabolites and other bioactive molecules, thereby influencing recipient-cell function (44). Their membrane-enclosed architecture and capacity to carry biologically active cargo have also prompted considerable interest in their use as therapeutic delivery vehicles (45, 46). The study of EVs should, however, be guided by standardized nomenclature and reporting criteria. MISEV2023 defines EVs as lipid-bilayer-delimited extracellular particles and recommends distinguishing them from non-vesicular extracellular particles (NVEPs), which may co-isolate during EV preparation. It further emphasizes transparent reporting of sample collection, separation and concentration procedures, physicochemical and molecular characterization, and functional and *in vivo* studies. These considerations are particularly relevant to bacterial EVs, whose composition and heterogeneity can vary with bacterial species, strain and growth conditions and whose preparations may contain non-vesicular bacterial components or EVs from other sources (47). Before considering engineering strategies, it is therefore important to define the source, nomenclature and characterization of microbiota-derived EVs, as these parameters directly influence the interpretation of their biological activity and therapeutic potential.

### 5.1 Structure and classification of gut microbiota-derived EVs

EVs of gut microbiota origin have been recognized as a new dialogue medium between the gut and bone. Gut microbiota-derived EVs are functional nanovesicles secreted by various microbial species in the intestinal tract, containing a diverse repertoire of biomolecules—including functional proteins, nucleic acids, and lipids—from their parental bacteria, and have attracted widespread attention for their potential to modulate host immunity and metabolism (48).

Based on their parental origin, EVs of gut microbiota origin can be classified into two major groups (45): (1) EVs from Gram-negative bacteria, also known as outer membrane vesicles (OMVs), which originate from the outer membrane and carry components from the periplasm and cytoplasm, with diameters ranging from 20 to 250 nm; and (2) EVs from Gram-positive bacteria, also known as cytoplasmic membrane vesicles (CMVs), which originate from the cytoplasmic membrane and carry cytoplasmic material, with diameters ranging from 20 to 400 nm. Through in-depth characterization of the molecular components carried within EVs and their functions, the mechanisms of microbial-host interactions can be elucidated, thereby revealing new avenues for intervening in microbial community-host crosstalk (49). The growing body of EV research opens up new possibilities for therapeutic strategies and provides an innovative platform for individualized healthcare.

### 5.2 EVs derived from gut microbiota regulate the gut-bone axis

EVs can carry growth factors, signaling molecules, or functional proteins to modulate OC differentiation and bone remodeling. The functional effects of microbiota-derived EVs are mediated by a diverse repertoire of protein cargoes, as summarized in Table 6.

**Table 6:** Representative EV-associated proteins and their reported effects on bone and inflammatory processes.

EVs - related Proteins	Sources	*In vitro*	*In vivo*	References
DnaK	Natural strain	Down - regulates the expression of NFATc1 and CTSK and inhibits OC differentiation	Promotes the apoptosis of peripheral blood T cells and the M1 polarization of liver macrophages in mice	(85)
BMP-2, CXCR4	Engineered strains	Up-regulation of the expression of osteogenesis-related genes (e.g., Runx2, OCN, OPN) significantly promotes osteogenic differentiation and inhibits adipogenic differentiation in BMSCs	BMP-2 and CXCR4 fusion BEVs have good bone targeting properties and significantly improve OP symptoms in OVX mice, promoting bone formation through activation of the BMP/SMAD signaling pathway	(54)
LTA	Natural strain	Confirmation that EVs are derived from *Lactobacillus salivarius* and are structurally intact	EVs were able to enter bone tissue, repair the intestinal barrier function, reduce serum LPS levels, and improve OP symptoms in ORX rats.	(86)
MLCK, ZO-1, Occludin	Natural strain	——	Oral administration of *Lactobacillus salivarius* and its EVs decreases MLCK expression, increases ZO-1 and Occludin expression, repairs intestinal mucosal barrier damage, and improves OP symptoms in ORX rats	(86)
LGG-EVs	Natural strain	——	Prevention of colonic tissue damage and colon shortening, improvement of intestinal inflammation, and inhibition of TLR4-NF-κB-NLRP3 axis activation	(87)
LrEVs	Natural strain	It can be taken up by chicken macrophages, inhibit pro-inflammatory response and enhance anti-inflammatory response.	Ameliorate LPS-induced decline in broiler growth performance, reduce mortality, attenuate intestinal damage, and reduce inflammatory response by regulating cytokine gene expression, inhibit LPS-induced MPO activity	(88)
BEVs	Natural strain	Promotes phagocytosis by macrophages and enhances bactericidal activity.	Reduces mortality, reduces organ damage, inhibits inflammation, and reduces bacterial burden in a mouse model of sepsis	(89)
*Akkermansia muciniphila* EVs	Natural strain	Enhances intestinal integrity of cell lines by up-regulating tight junction genes, activating TLR-2 expression while decreasing TLR-4 expression	Reduces body weight, reduces adipose inflammation, improves HFD-induced intestinal barrier dysfunction, and affects the expression of fat storage-related gene ANGPTL4 in obese mice	(90)
LjEVs	Natural strain	Inhibition of macrophage MAPK signaling pathway and enhancement of M2 macrophage polarization lead to repair of the intestinal barrier	Relief of diarrhea symptoms and intestinal inflammatory damage, a process dependent on macrophages	(91)
LP-UJS-EVs	Natural strain	Promotes intestinal epithelial cell growth and induces macrophage M2 polarization	Relieve UC symptoms, repair intestinal barrier, reduce intestinal inflammation, regulate gut microbiota, increase the abundance of beneficial bacteria *Prevotella*	(92)

DnaK, Chaperone protein DnaK; BMP - 2, Bone Morphogenetic Protein - 2; CXCR4, C - X - C chemokine receptor type 4; LTA, Lipoteichoic acid; MLCK, Myosin light - chain kinase; ZO - 1, Zonula occludens - 1; LGG - EVs, *Lactobacillus rhamnosus* GG - derived extracellular vesicles; LrEVs, *Lactobacillus reuteri* - derived extracellular vesicles; BEVs, Bacterial extracellular vesicles; LjEVs, *Lactobacillus johnsonii*- derived extracellular vesicles; LP-UJS-EVs, *Lactobacillus plantarum* UJS001 - derived extracellular vesicles; NFATc1, Nuclear factor of activated T - cells; CTSK, Cathepsin K; OPN, Osteopontin; TLR2, Toll - like receptor 2; TLR4, Toll - like receptor 4; MAPK, Mitogen - Activated Protein Kinase; BMP/SMAD, Bone Morphogenetic Protein/Small Mothers Against Decapentaplegic; LPS, Lipopolysaccharide; ORX:orchiectomy; NF - κB, Nuclear Factor - kappa B; NLRP3, Nucleotide - binding oligomerization domain - like receptor family, pyrin domain - containing 3; MPO, Myeloperoxidase; HFD, High - Fat Diet; ANGPTL4, Angiopoietin - like protein 4; UC, Ulcerative Colitis.

Wang et al. found that OMVs from the Gram-negative bacterium *Proteus mirabilis* inhibited OC differentiation and bone resorption by regulating the miR-96-5p/ABCA1 axis, thereby ameliorating bone loss in both OP and collagen-induced arthritis models (50). This finding illustrates that vesicles derived from a pathogenic bacterium can exert context-dependent osteoprotective effects, despite the pathogenic potential of their parent organism. Mechanistically, *P. mirabilis* OMVs promoted mitochondrial dysfunction and mitochondria-dependent apoptosis in osteoclasts, with the miR-96-5p/ABCA1 axis implicated in this response. However, the specific OMV cargo responsible for initiating this pathway remains unresolved. This example also highlights an important translational safety consideration. As *P. mirabilis* is a pathogenic Gram-negative bacterium, its OMVs may contain LPS and other pathogen-associated molecular patterns (PAMPs), which could induce unwanted inflammatory or immunotoxic responses. Notably, purified *P. mirabilis* LPS did not reproduce the effects of the OMVs on osteoclastogenesis under the conditions tested, suggesting that the observed osteoprotective activity cannot be attributed to LPS alone. Nevertheless, the presence of endotoxin and other bacterial components remains a relevant safety concern. Importantly, the experimental intervention involved isolated OMVs rather than administration of live *P. mirabilis*, indicating that a vesicular therapeutic product can be physically separated from the pathogenic organism itself. Such separation, however, does not eliminate pathogen-associated molecules carried by the vesicles. Therefore, future development of *P. mirabilis*-derived or other pathogenic-bacterium-derived EVs should prioritize stringent purification, endotoxin control, comprehensive cargo characterization, and dedicated *in vivo* safety assessment.

In a separate study, ultrasound-activated probiotic OMV coatings were shown to treat implant-associated infections through bacterial cuproptosis-like death and immunomodulation when applied to peptide skeleton implants for bone defect repair (51). Furthermore, transplantation of pediatric-derived *Enterococcus faecalis* EVs (CGM-EVs) was shown to reverse OVX-induced bone loss by restoring the abundance of *Akkermansia muciniphila* (20). Restoration of Akk levels independently repaired OVX-induced disturbances in bone metabolism, thereby treating OP. This osteoprotective effect was attributed to the secretion of Akk-OMVs, which infiltrate bone tissue, enhance osteogenic activity, and inhibit OC formation (20) (Figure 3).

**Figure 3:**
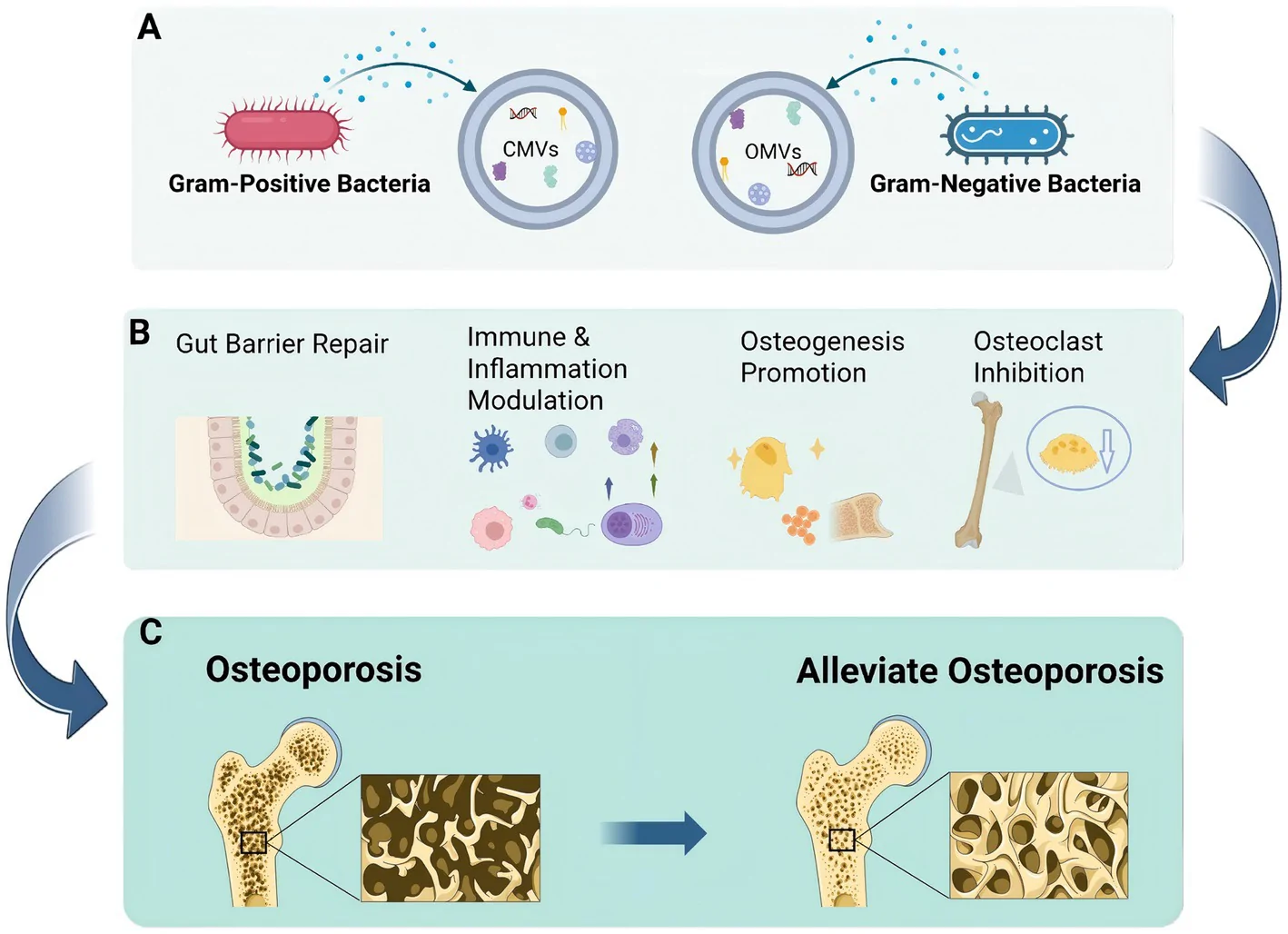
Schematic diagram of the mechanism by which gut bacterial EVs regulate OP via the gut-bone axis. **(A)** Gram-positive and Gram-negative bacteria secrete CMVs and OMVs, respectively, as signal mediators between bacteria and the host. **(B)** BEVs regulate bone metabolic balance through four pathways: repairing the gut barrier, modulating immune inflammation, promoting OB differentiation, and inhibiting OC activation. **(C)** BEVs intervention reverses the pathological bone loss in OP, characterized by increased trabecular bone volume, improved trabecular connectivity, and enhanced bone mineral density, thereby effectively alleviating OP. BEVs: bacterial extracellular vesicles.

While natural microbiota-derived EVs show clear osteoprotective activity in preclinical models, their translational potential is constrained by three inherent limitations: poor bone-targeting specificity, rapid clearance in circulation, and batch-to-batch variability in cargo composition. These challenges are not unique to bacterial EVs—they mirror the barriers faced by mammalian EV therapeutics—but they are amplified by the additional complexity of microbial source material and the presence of immunostimulatory components such as lipopolysaccharide (LPS). These limitations have motivated the development of engineered EV platforms that incorporate targeting moieties, therapeutic payloads, and biomaterial carriers to enhance efficacy, safety, and manufacturability. The following section surveys the major engineering strategies currently being explored to address these translational bottlenecks.

### 5.3 Bone-targeted engineered EV platforms and translational considerations

Studies have increasingly demonstrated that EVs can serve as natural carriers of osteogenic bioactive molecules, mimic certain functional properties of the extracellular matrix, and facilitate intercellular communication among bone formation-related cells, thereby contributing to the maintenance of bone homeostasis and the promotion of bone regeneration (52) (Figure 4). Owing to their intrinsic biocompatibility, nanoscale size, and cargo-delivery capacity, EVs have attracted considerable attention as promising therapeutic platforms for OP.

**Figure 4:**
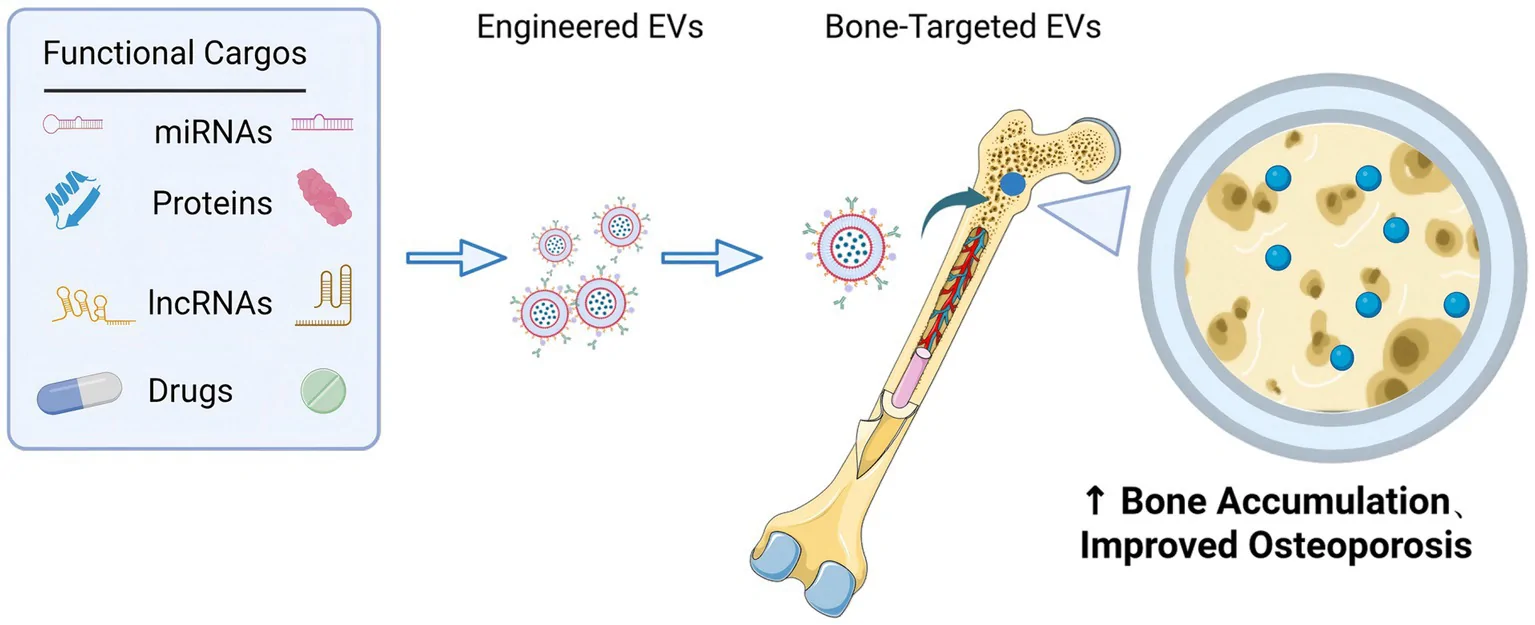
Schematic diagram of bone-targeting engineered EVs for OP therapy. This diagram illustrates the strategy of engineered EVs for targeted OP treatment. Functional cargos (including miRNAs, proteins, lncRNAs, and drugs) are loaded into EVs, followed by surface engineering to generate bone-targeting EVs. These modified EVs specifically accumulate in bone tissue, exerting local therapeutic effects by promoting osteogenesis and inhibiting bone resorption, thereby improving OP. miRNAs: microRNAs; lncRNAs: long non-coding RNAs.

Building on this concept, researchers have engineered recombinant probiotic-derived EVs with both bone-targeting and osteogenesis-promoting properties using the strain *Escherichia coli* Nissle 1917 carrying the pET28a-ClyA-hCXCR4 construct (53). This strategy provided an important basis for the development of functionalized EVs with enhanced tissue specificity. Subsequently, a recombinant *E. coli* Nissle 1917-pET28a-ClyA-BMP-2-CXCR4 strain was constructed to generate engineered EVs simultaneously enriched with osteogenic and bone-targeting components. In this design, bone morphogenetic protein-2 (BMP-2), a key regulator that promotes OB differentiation and bone formation, and CXCR4, a bone-targeting molecule, were fused with the EV surface protein ClyA and overexpressed on the vesicle surface (54). Through this synthetic biology-based modification, more efficient bone-targeted therapeutic engineered EVs, termed BEVs-BMP-2-CXCR4, were successfully obtained. Parallel to synthetic biology approaches, chemical conjugation offers a simpler and faster route for EV surface modification without genetic manipulation. For example, thiol-click chemistry has been used to attach the bone-targeting peptide SDSSD to LGG-EVs (BT-LGG-EVs), enhancing hydroxyapatite affinity and bone accumulation (53). Physical loading techniques, including electroporation, sonication, and extrusion, enable efficient encapsulation of diverse therapeutic cargoes—such as SOST siRNA and osteogenesis-related miRNAs—into preformed EVs, although these methods inevitably compromise membrane integrity to some extent (55). Biomaterial-assisted delivery further extends EV therapeutic potential by incorporating EVs into hydrogels or ECM-mimetic scaffolds, achieving sustained local release at bone defect sites. These complementary delivery strategies are compared in Table 1.

Notably, these engineered EVs not only retained the inherent advantages of natural vesicles as delivery vehicles but also exhibited enhanced targeting capability and improved therapeutic efficacy. By precisely delivering bone-protective and osteogenesis-related proteins to bone tissue, BEVs-BMP-2-CXCR4 effectively inhibited bone loss and significantly ameliorated OVX-induced OP *in vivo* (54). These findings highlight the substantial potential of integrating synthetic biology with EV engineering to develop next-generation therapeutics for OP.

While these engineering platforms offer promising therapeutic efficacy, their clinical translation is critically dependent on a thorough understanding of their safety and immunotoxicological profiles. The therapeutic efficacy of engineered EVs is further augmented by the selection of appropriate cargoes. Beyond osteogenic proteins such as BMP-2, bioactive peptides and small interfering RNAs (siRNAs) have been successfully loaded into BEVs for synergistic anti-osteoporotic effects. For instance, FRATtide—a peptide that inhibits the Akt/GSK-3β/NFATc1 pathway—has been encapsulated in BEV-DCS to suppress osteoclastogenesis (56). Similarly, SOST siRNA delivered via bone-targeted BEVs silences sclerostin expression, thereby activating Wnt/β-catenin signaling and promoting bone formation (55). These diverse cargo types, along with their mechanisms and therapeutic outcomes, are summarized in Table 7.

**Table 7:** Therapeutic cargoes delivered by engineered EVs for osteoporosis.

Cargo type	Representative cargo	Therapeutic target	Mechanism of action	Effect on osteoporosis	References
siRNA	SOST-siRNA	OB	SOST silencing activates Wnt/β-catenin signaling	Promotes bone formation	(55)
Bioactive peptide	FRATtide	Pre-OCs	Inhibits Akt/GSK3β/NFATc1 pathway	Suppresses osteoclastogenesis	(56)
Protein	BMP2	MSCs/OB	Activates BMP/Smad signaling	Enhances osteogenesis	(54)
Endogenous miRNAs	Osteogenesis-related miRNAs	OB lineage cells	Regulate osteogenic gene expression and differentiation pathways	Promote bone formation and mineralization	(59)
Regulatory proteins	Pro-regenerative proteins	Bone microenvironment	Modulate osteogenesis, angiogenesis and immunoregulation during bone repair	Improved bone regeneration potential	(59)

siRNA, Small Interfering RNA; SOST, Sclerostin; Wnt, Wingless-type (signaling pathway); BMP2, Bone Morphogenetic Protein 2; SMAD, Small Mothers Against Decapentaplegic; Akt, Protein Kinase B (also known as AKT); GSK3β, Glycogen Synthase Kinase 3 Beta; NFATc1, Nuclear Factor of Activated T-cells, Cytoplasmic 1; MSCs, Mesenchymal Stem Cells; miRNA, MicroRNA; OP, Osteoporosis.

#### 5.3.1 Safety and immunotoxicological considerations of bacteria-derived EVs

Beyond their therapeutic potential, the clinical translation of bacteria-derived EVs must be critically weighed against their inherent safety liabilities. Bacterial EVs naturally carry pathogen-associated molecular patterns (PAMPs), including LPS in Gram-negative bacteria and lipoteichoic acid (LTA) in Gram-positive bacteria, which can activate Toll-like receptor (TLR) pathways and trigger innate immune responses. While moderate immune stimulation may be therapeutically beneficial in certain contexts—such as enhancing anti-infective immunity—repeated or systemic administration poses significant risks of hyperinflammation, including cytokine storms (e.g., sharp elevations in TNF-α and IL-6), endotoxemia-like reactions, and potential induction of anti-EV antibodies that accelerate vesicle clearance and abrogate therapeutic efficacy.

Importantly, many current *in vivo* studies rely primarily on body weight changes and H&E staining of major organs for toxicity assessment, with limited systematic evaluation of: (1) time-course cytokine profiles following administration; (2) anti-EV antibody generation under repeated dosing regimens; (3) chronic toxicities associated with EV accumulation in the reticuloendothelial system (liver, spleen, and lungs); or (4) Potential perturbations of the resident gut microbiota following EV exposure also warrant further investigation. Moreover, although genetic engineering approaches to attenuate LPS-associated immunostimulation may improve the safety profile of BEVs, the incorporation of exogenous functional proteins, such as BMP-2 or CXCR4, could potentially alter the immunogenicity of engineered vesicles by introducing novel antigenic determinants and thereby increasing the risk of anti-drug antibody (ADA) responses. However, this potential immunogenicity has received relatively limited attention in the current BEV literature. To date, systematic evaluation of repeated-dose immunogenicity remains limited, particularly for long-term administration. We therefore emphasize that even engineered bacterial EVs should not be assumed safe by default; rigorous immunogenicity profiling, LPS quantification (e.g., LAL or recombinant factor C assays), TLR activation reporter assays, and biodistribution tracking in aged, osteoporotic, and comorbid animal models must be mandated as obligatory components of preclinical safety evaluation before any clinical advancement can be responsibly pursued. For translational purposes, local administration (e.g., intraosseous injection or local delivery at bone defect sites) may represent an alternative strategy to reduce systemic exposure and warrants further investigation.

#### 5.3.2 Critical comparison of engineering platforms and remaining challenges

While synthetic biology-based surface display (e.g., the ClyA-fusion system) offers genetic stability and scalable production, its reliance on live bacteria raises biosafety containment concerns, and the insertion of exogenous proteins may provoke anti-drug antibody responses, as discussed above (54). Chemical conjugation methods, such as thiol-click chemistry for bone-targeting peptide (SDSSD) attachment, provide a simpler and faster modification route without genetic manipulation; however, the conjugation efficiency is often suboptimal, and the long-term stability of surface-modified peptides under physiological conditions remains uncertain (53). Physical loading techniques (electroporation, sonication, extrusion) are versatile for encapsulating diverse cargoes such as siRNA and miRNAs, but they inevitably compromise EV membrane integrity, leading to cargo leakage and batch-to-batch variability. Biomaterial-assisted delivery (e.g., hydrogels) significantly improves local retention at bone defects, yet its application is largely restricted to focal bone repair rather than systemic OP.

Critically, most engineering studies have only been validated in acute OVX models (typically 4–8 weeks post-ovariectomy), with no data on whether these modifications alter EV biodistribution, clearance kinetics, or long-term toxicity in aged, osteoporotic, or comorbid animal models that better recapitulate the human condition. Furthermore, direct head-to-head comparisons of these platforms with respect to therapeutic efficacy, safety, and manufacturability remain limited, making it difficult to determine which platform is best suited to specific clinical scenarios. We therefore advocate for standardized reporting of engineering parameters, rigorous immunogenicity profiling, and side-by-side validation under harmonized preclinical conditions. These measures are essential prerequisites for advancing these technologies toward clinical translation.

## 6 Conclusion and perspective

OP, as a systemic metabolic bone disease with a continuously rising incidence driven by global population aging, has become a major public health challenge that endangers the health of the elderly and postmenopausal women, while imposing a heavy economic and social burden on families and society. Conventional anti-OP therapeutic agents, broadly divided into antiresorptive and anabolic therapies, remain the cornerstone of OP management and have demonstrated robust efficacy in reducing fracture risk. Nevertheless, treatment selection and long-term management require careful consideration of individual fracture risk, treatment duration, adherence and the balance between efficacy and safety. In this context, the discovery of the “gut–bone axis” regulatory network has expanded the traditional research framework of OP pathogenesis and treatment, and the gut microbiota, as an important regulator of this axis, has emerged as a promising target for OP intervention, providing a potential complementary avenue for the development of novel strategies for the prevention and management of OP.

Beyond dissecting the individual mechanistic pathways discussed above, the future of OP management lies in translating these molecular insights into an actionable, closed-loop clinical strategy. To illustrate this integrated paradigm, we present a conceptual roadmap that encapsulates the multi-step pipeline from patient stratification to dynamic therapeutic modulation (Figure 1). This framework bridges the gut-bone axis with precision medicine, highlighting how multi-omics profiling and AI-driven prediction bifurcate into two complementary intervention modalities—namely, personalized probiotics and engineered bacterial EVs—before converging on bone-targeted delivery and long-term disease management.

In conclusion, accumulating evidence has demonstrated that the gut microbiota plays a crucial role in the regulation of bone remodeling and the pathogenesis of OP through the “gut-bone axis.” This review synthesizes current evidence on how the gut microbiome and probiotics influence bone homeostasis. These factors can directly modulate OB and OC differentiation and function through microbial metabolites and EVs, while also indirectly influencing skeletal homeostasis by maintaining intestinal barrier integrity, regulating immune and inflammatory responses, and modulating endocrine signaling. These findings indicate that the gut microbiota is not only an important component of systemic metabolic regulation, but also a promising target for the prevention and treatment of OP.

Among the diverse mediators involved in gut-bone communication, gut microbiota-derived EVs have attracted increasing attention because of their unique biological properties, including nanoscale size, natural biocompatibility, cargo-carrying capacity, and potential for functional engineering. Current studies suggest that these EVs can promote osteogenesis, inhibit osteoclastogenesis, repair intestinal barrier dysfunction, and reshape the immune microenvironment, thereby exerting beneficial effects on bone metabolism. In particular, the development of engineered EVs through synthetic biology has further expanded the translational potential of this field by enabling the targeted delivery of osteogenic and anti-resorptive molecules to bone tissue.

However, despite these advances, several critical challenges remain to be addressed. First, the precise bioactive components within microbiota-derived EVs that mediate anti-osteoporotic effects have not yet been fully elucidated. Although proteins, nucleic acids, lipids, and metabolites carried by EVs are all likely to contribute to their biological functions, the specific roles of EV-associated functional proteins in regulating OB and OC differentiation and function remain largely unclear. Second, the mechanisms by which gut microbiota-derived EVs reach skeletal tissues and participate in the regulation of the bone microenvironment require further investigation. In addition to OBs and OCs, bone remodeling also involves osteocytes, bone lining cells, osteomacs, vascular endothelial cells, and other components within the basic multicellular unit, all of which may represent potential targets of EV-mediated regulation.

Furthermore, several translational barriers must be overcome before EV-based strategies can be applied clinically. These include the optimization of EV production and purification, improvement of cargo-loading efficiency, enhancement of tissue-targeting specificity, and establishment of standardized systems for quality control, biosafety evaluation, and large-scale manufacturing. Moreover, the biological heterogeneity of EVs derived from different microbial strains highlights the need for more rigorous comparative studies and standardized evaluation criteria.

An additional layer of complexity arises from inter-individual variability in gut microbiota composition and therapeutic responses. Host-specific factors, including age, sex, dietary patterns, comorbidities, and concomitant medications, may shape the gut microbial ecosystem and influence probiotic engraftment and treatment responses (57, 58). Beyond compositional differences, functional heterogeneity within the gut microbiome—including differences in SCFA production and bile acid metabolism—may further influence the bioavailability, cargo composition, and biological activity of probiotic-derived EVs. Host genetic variation may represent an additional determinant of therapeutic response; for example, polymorphisms in innate immune receptors such as TLR4 have been associated with differential responses to bacterial PAMPs and could potentially affect the safety–efficacy balance of EV-based interventions. Collectively, these host–microbiome interactions underscore the need for stratified clinical trial designs that integrate baseline microbiome features with relevant host characteristics to identify determinants of treatment response. In the longer term, such approaches may provide a foundation for microbiome-guided precision strategies, including rational strain selection and individualized EV-based interventions. Sex-specific responses also warrant greater attention, as most preclinical OP studies have focused on ovariectomy-induced bone loss, whereas male OP and androgen-deficiency-associated mechanisms remain comparatively understudied.

Among these priorities, we consider the identification of EV-associated functional proteins that simultaneously promote osteogenesis and inhibit bone resorption to be the most urgent, as it would provide both mechanistic insight and a defined quality-control marker for EV-based therapeutics. Equally pressing is the need for standardized safety evaluation frameworks—including immunogenicity profiling, LPS quantification, and biodistribution tracking in aged, osteoporotic, and comorbid animal models—before clinical translation can be responsibly pursued.

Looking forward, future studies should integrate multi-omics technologies with functional validation to dissect the molecular mechanisms underlying EV-mediated gut-bone communication. In parallel, the rational design of bone-targeted engineered EVs—combining synthetic biology with chemical conjugation, physical loading, or biomaterial-assisted delivery—may offer a precision toolbox for bone metabolic disorders. Ultimately, further exploration of microbiota-derived EVs, particularly their functional protein cargos and engineering potential, will not only deepen our understanding of the gut-bone axis but also accelerate the development of safe, effective, and targeted therapeutic strategies for OP.
